# Editorial for the Special Issue “Selected Papers from the 3rd International Electronic Conference on Microbiology (ECM 2025)”

**DOI:** 10.3390/microorganisms14040793

**Published:** 2026-03-31

**Authors:** Nico Jehmlich

**Affiliations:** Department of Molecular Toxicology, UFZ-Helmholtz Centre for Environmental Research, 04318 Leipzig, Germany; nico.jehmlich@ufz.de

This Special Issue, “Selected Papers from the 3rd International Electronic Conference on Microbiology (ECM 2025),” brings together six papers that reflect the broad and interdisciplinary nature of microbiology. The articles cover environmental, clinical, and molecular aspects of microbial research and highlight how emerging technologies and integrative approaches are advancing our understanding of microbial systems. Recent developments in the field of microbiology have made significant progress in molecular diagnostics, microbiome research, and systems biology, as illustrated by the review on cultivation and excystation strategies for the protozoan parasite *Giardia lamblia* [1].

Contributions in this collection address clinically relevant aspects of microbiology. Antimicrobial resistance remains a central topic, and new strategies targeting bacterial DNA repair mechanisms, such as inhibition of the RecA protein, have been proposed as promising approaches to mitigate resistance development [2]. In addition, metabolic responses of pathogenic bacteria to antibiotic exposure are being investigated, with compounds such as phenyllactic acid being proposed as potential metabolic markers for antibiotic-induced activity in nosocomial strains of *Klebsiella pneumoniae* [3].

Host–microbe interactions also represent a key research focus. Viral infections can influence inflammatory signalling pathways and immune responses, as demonstrated for human herpesvirus 6 [4]. In parallel, microbiome-based approaches are increasingly being used to explore links between microbial community composition and metabolic health. For example, microbial signatures associated with normal and elevated blood glucose levels have been investigated to better understand potential links between microbiome composition and metabolic health [5].

Environmental and agricultural microbiology are also represented in this Special Issue. Research on indigenous bacteria from olive orchard soils demonstrates the potential of native microbial communities as biological control agents and highlights the importance of soil microbiome analyses to identify beneficial microorganisms [6].

Together, these contributions show how different research areas are becoming more interconnected. However, despite this progress, important gaps remain. Many studies are still descriptive. We need more functional validation, a clearer understanding of mechanisms, and stronger links between laboratory results and real-world applications. Future research should combine multi-omics approaches with experimental studies. Moreover, better standardization and stronger translation into clinical and environmental practice are also needed. Overall, this Special Issue reflects the dynamic research presented at ECM 2025 and highlights promising paths for advancing microbiology through innovation and collaboration.
